# Faculty appointments and the record of scholarship

**DOI:** 10.7554/eLife.00452

**Published:** 2013-01-08

**Authors:** Amy Brand

**Affiliations:** is Assistant Provost for Faculty Appointments and Information at Harvard University, where she manages the review of faculty appointments University-wide. Her career spans academia, publishing and scholarly communication technologies. She was program manager of the Harvard Office for Scholarly Communication from 2008 to 2009, and before that held long-term positions as an executive editor at the MIT Press, and as director of business and product development at CrossRef. She is also on the board of directors for ORCID.amy_brand@harvard.edu

**Keywords:** Point of view, publishing, academic career, contributorship, ORCID

## Abstract

Academic review committees would benefit from more details about the contributions made by individual researchers to papers with multiple authors, and also from more information about other types of scholarly communication.

Have you noticed that conversations about scholarly communication and academic careers often conclude with someone saying: ‘Well, until university provosts flip the switch and start taking papers in open-access journals, the publication of data and code, digital monographs and so on into account when making decisions about promotion and tenure, traditional publications and metrics will continue to hold us all hostage’?

I have lost count of the number of times I've heard arguments to this effect at academic library and publishing conferences, and I confess it is part of what drew me toward my current post as assistant provost for faculty appointments and information at Harvard. If the provost's office is where change in the dissemination of scholarship can be ignited, that's where I want to be.

With a few years of first-hand experience in the administration of faculty appointments under my belt, I can report back—from the inside, as it were—that there is no such switch. It is much more complicated than a consensus on the part of university provosts to expand the set of works and measures that constitute solid evidence of academic distinction. That said, there is growing awareness that the search and review committees that appoint and promote academic staff have traditionally relied on information sources that may fail to portray the full picture. This is especially true when the candidate's key contributions are not published in book or journal form, or when he/she publishes mainly or exclusively co-authored works.

## The importance of peers

It would be imprudent to generalize about Harvard's criteria for the appointment and promotion of faculty since the policies and procedures of the different Schools within the University all differ to a degree. And as Richard Zare, former chair of the chemistry department at Stanford University, described in a recent article (Zare, 2012), elite research institutions in the United States, seemingly unlike most of the world's universities, are not overly dependent on citation-based measures of impact such as impact factors for journals or h-indices for individuals. Rather, their appointment processes are time-intensive, more qualitative than quantitative or formulaic, and rely most heavily on peer review (both internal and external) to identify excellence. This type of peer review involves collecting confidential letters from a well-chosen set of peers: the purpose of these letters is to help academic review committees assemble a robust qualitative picture of a scholar's originality, independence, intellectual leadership and potential for future productivity and impact.

In 2010, *Nature* carried out a survey in which it asked readers about the use of metrics in decisions about new hires and tenure (Abbott et al., 2010). Three-quarters of the 150 readers who replied thought that metrics were being used in hiring decisions. However, provosts and other administrators contacted by *Nature* painted a different picture: ‘Metrics are not used a great deal,’ said Alex Halliday, head of the mathematical, physical and life sciences division at Oxford University. ‘The most important things are the letters, the interview and the CV, and our opinions of the papers published.’ Claude Canizares, vice president for research and associate provost at the Massachusetts Institute of Technology, had a similar message: ‘We pay very little attention, almost zero, to citation indices and counting numbers of publications’.

While the letters that inform faculty appointments usually succeed in bringing the key considerations to light, I believe we could make the work of search and review committees easier and more reliable if we enhanced our sources of information about scholarship and contribution in ways that I describe below.

We could make the work of search and review committees easier and more reliable if we enhanced our sources of information about scholarship and contribution.

## A broader record of scholarship

When a scholar's portfolio includes works outside of the published record of books, journal articles and conference proceedings, search and review committees often have to dig deeper to gauge the academic impact of those works. In the visual arts, for example, they may consult exhibition histories and published critiques, in addition to external reviews from a wider set of peers that might include curators and fellow artists. In the sciences, a candidate may have contributed meaningfully to his or her field by making data or software code widely available. In such cases, there is no choice at present but to rely on peer testimony to weigh the significance of the contribution.

**Figure 1.**
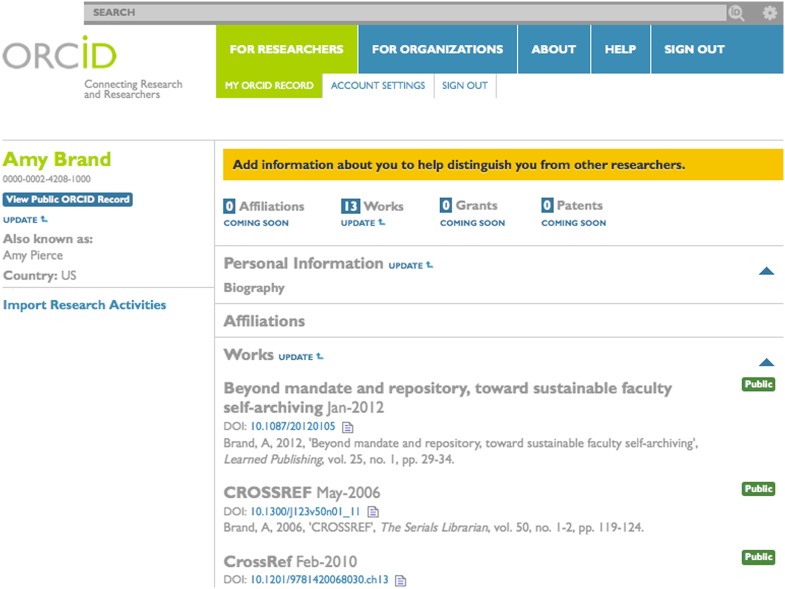
The ORCID (Open Researcher and Contributor ID) initiative aims to provide every researcher in the world with a unique identifier linked to his or her academic outputs and activities. This figure shows Amy Brand's ORCID page.

Researchers and review committees alike would benefit greatly from the development of standards for the identification and citation of an extended set of scholarly works including data, software and code, multimedia and internet communications, so that such contributions are more readily integrated into our evolving digital scholarly ecosystem. The just-launched ORCID network [http://www.orcid.org], which aims to provide every researcher in the world with a unique identifier linked to academic outputs and activities (see Figure 1), may provide a new way for scholars to associate themselves within this ecosystem with a wider array of scholarly contributions. In the sciences, standard methods for citing data and attributing credit for the generation of quantitative data are near-term priorities (Altman and King, 2007; National Research Council, 2012).

## Contributorship versus authorship

For the purposes of academic review, the traditional publication list is especially problematic when it comes to scholars with many multi-authored works. This is because author lists don't provide clear information about who contributed what in large-scale research and writing collaborations, which are increasingly common in the physical and life sciences. Author lists take a complex network of collaboration and effectively reduce it to a one-dimensional information source, where all we have to go by is order of names to convey author role and rank. Different fields of scholarship have adopted different practices around author order, but middle authors are always at the greatest risk of losing out on attribution and credit because their *perceived* contribution is diminished relative to first and last authors in collaborative works. In fields that list author names in journal articles alphabetically, such as economics, it has even been observed that you are more likely to get tenured in a top department if your surname begins with a letter earlier in the alphabet (Einav and Yariv, 2006). The unintended consequences of our authorship practices are far from benign for researchers and for science.

There is growing interest among researchers, funding agencies, academic institutions, editors and publishers in increasing the transparency of research contributions, and in more fine-grained tracking of attribution and associated credit. Many publishers now require contribution disclosures upon article submission—some in structured form, some in free-text form. There is now a clear need for a standard vocabulary of contributor roles that captures what each named author contributed to a particular publication—for example, conceptual framework, methodological design, data collection, data curation, experimental procedures, programming, software, statistical analysis, investigation, instrumentation, writing, illustration, project management, funding, laboratory head and so on. With the development of contributor role vocabularies and tagging mechanisms, the review committee of the future reading a tenure application from, say, a biostatistician who is usually a middle author in multi-authored works, will more readily be able to detect that the individual consistently contributes innovative methodologies to his or her research collaborations (IWCSA, 2012).

Imagine how the academic appointment process might change if search and review committees had access—within an appropriately tagged or linked online CV, for example, or via the ORCID system—to information about the specific contributions made by a candidate to each of his/her works, including contributions that might not otherwise have qualified for ‘authorship’ status? While a future scheme of standardized contributor roles integrated with the ORCID identifier system may lead to the development of new quantitative metrics, I am much more interested in having more precise information about researcher contribution made transparent in our information resources. On-going developments in academic publishing, along with the semantic capabilities of the web, should make this possible before too long.
